# Activated Platelets from Diabetic Rats Cause Endothelial Dysfunction by Decreasing Akt/Endothelial NO Synthase Signaling Pathway

**DOI:** 10.1371/journal.pone.0102310

**Published:** 2014-07-21

**Authors:** Keiko Ishida, Kumiko Taguchi, Takayuki Matsumoto, Tsuneo Kobayashi

**Affiliations:** Department of Physiology and Morphology, Institute of Medicinal Chemistry, Hoshi University, Shinagawa-ku, Tokyo, Japan; Max-Delbrück Center for Molecular Medicine (MDC), Germany

## Abstract

Diabetes is associated with endothelial dysfunction and platelet activation, both of which may contribute to increased cardiovascular risk. The purpose of this study was to characterize circulating platelets in diabetes and clarify their effects on endothelial function. Male Wistar rats were injected with streptozotocin (STZ) to induce diabetes. Each experiment was performed by incubating carotid arterial rings with platelets (1.65×10^7^ cells/mL; 30 min) isolated from STZ or control rats. Thereafter, the vascular function was characterized in isolated carotid arterial rings in organ bath chambers, and each expression and activation of enzymes involved in nitric oxide and oxidative stress levels were analyzed. Endothelium-dependent relaxation induced by acetylcholine was significantly attenuated in carotid arteries treated with platelets isolated from STZ rats. Similarly, treatment with platelets isolated from STZ rats significantly reduced ACh-induced Akt/endothelial NO synthase signaling/NO production and enhanced TXB_2_ (metabolite of TXA_2_), while CD61 (platelet marker) and CD62P (activated platelet marker) were increased in carotid arteries treated with platelets isolated from STZ rats. Furthermore, the platelets isolated from STZ rats decreased total eNOS protein and eNOS dimerization, and increased oxidative stress. These data provide direct evidence that circulating platelets isolated from diabetic rats cause dysfunction of the endothelium by decreasing NO production (via Akt/endothelial NO synthase signaling pathway) and increasing TXA_2_. Moreover, activated platelets disrupt the carotid artery by increasing oxidative stress.

## Introduction

Diabetes mellitus is associated with the accelerated development of cardiovascular diseases which are the primary cause of morbidity and mortality in patients [1]–[4]. Indeed, it has been well established that the pathogenesis and progression of vascular complications of diabetes are characterized by the development of endothelial dysfunction, which correlates with a decrease in vasodilator factor release, such as nitric oxide (NO) or prostacyclin, as well as an increase in vasoconstrictor production, such as Thromboxane A_2_ (TXA_2_) [1], [5]–[8]. NO generated from endothelial NO synthase (eNOS) plays a key role in vascular homeostasis. Besides its vasodilatory effects on smooth muscle cells, NO inhibits the proliferation and migration of these cells and can regulate vascular remodeling. Numerous studies have demonstrated that Akt is important upstream of eNOS activation [9], [10]. At the cellular level and in isolated blood vessels, it has been well established that ACh increases NO production by activating the Akt signaling pathway that leads to eNOS phosphorylation [5]. In addition, several studies have shown that oxidative stress plays a determinant role in the reduced effect of endothelial NO and this may occur in the vasculature in diabetes [11]. Oxidative stress is known to reduce the biological activity of NO and generate deleterious metabolites such as peroxynitrite [12], [13].

Platelet (PLT) activation occurs in several cardiovascular diseases with reduced NO bioavailability, such as acute coronary syndrome [14], heart failure [3], insulin resistance [15], diabetes [16], [17], metabolic syndrome [18], and hypercholesterolemia [19], and may represent a key contributing factor in the process of atherosclerosis and its thrombonic complications. Interestingly, there are several lines of evidence that PLT inhibition has beneficial effects on the endothelial function and NO bioavailability [20], [21]. In the forearms of patients with symptomatic coronary artery disease it was demonstrated that PLT glycoprotein IIb/IIIa receptor blockade and PLT adenosine diphosphate receptor blockade prevented the development of atherosclerotic lesions [21] and improved endothelium-dependent vasodilation [20]. In addition, activated PLTs are important sources of reactive oxygen species such as superoxide anions, known to inactivate endothelium-derived NO [22]. Therefore, it is likely that an accommodation of PLT-endothelial interactions will play an important role in altering the endothelial function and NO bioavailability. However, few studies on endothelial dysfunction in diabetic states have directly assessed the relationship between PLT activation and endothelium-dependent relaxation.

A growing body of evidence indicates that PLT activation not only induces acute vascular thrombosis, but also has as yet unrevealed roles in vascular injury and the progression of atherosclerotic lesion formation [22]. Therefore, the present study was conducted to investigate the effects of activated PLTs from diabetic rats on endothelial dysfunction. Because the endothelial function can also be affected by diabetes, the present study assessed the effects and mechanisms by which PLTs isolated from STZ-induced diabetic rats affect NO bioavailability and oxidative stress in carotid arteries from the rats.

## Material and Methods

### Reagents

Streptozotocin (STZ), phenylephrine (PE), NG-nitro-L-arginine (L-NNA), nitroblue tetrazolium (NBT), and antibodies against β-actin were all purchased from Sigma Chemicals (St. Louis, MO, USA). Sodium nitroprusside (SNP) was from Wako (Osaka, Japan), while acetylcholine (ACh) was from Daiichi Pharmaceuticals (Tokyo, Japan). All other agents were dissolved in saline. All concentrations are expressed as the final molar concentration of the base in the organ bath. Horseradish peroxide (HRP)-linked secondary anti-mouse or anti-rabbit antibody was purchased from Promega (Madison, WI, USA). Antibodies against Akt, phosphorylated Akt at Ser^473^, phosphorylated eNOS at Ser^1177^, and CD61 were obtained from Cell Signaling Technology (Danvers, MA, USA), while the antibody against eNOS was from BD Bioscience (San Jose, CA, USA). The antibody against CD62P was from Abcam (Cambridge, MA, USA).

### Animals and experimental design

Experiments involved male Wistar rats that were 8-weeks old at the beginning of the study. The experimental design included two groups of rats – the first group without diabetes, and the second group with streptozotocin (STZ)-induced diabetes. Experimental diabetes was induced in randomly selected animals with a single injection via the tail vein of STZ at 65 mg/kg dissolved in citrate buffer, as reported previously [23], [24]. Age-matched control rats were injected with the buffer alone. Food and water were available ad libitum. The experiments described here were performed 28–44 weeks after the injection. The rats were euthanized with inhaled isoflurane and assigned to various experiments. The animal protocols were approved as conforming to the Guide for the Care and Use of Laboratory Animals by the issuing committee (Committee on the Care and Use of Laboratory Animals of Hoshi University, which is accredited by the Ministry of Education, Culture, Sports, Science, and Technology, Japan).

### PLT isolation

Blood for PLT isolation was collected via the abdominal aorta. Blood was collected in 3.8% trisodium citrate (w/v), centrifuged at 1,200 rpm (10 min, 20°C), and the top layer of PLT-rich plasma was removed. A PLT pellet was formed by centrifugation (3,000 rpm ×10 min; 20°C), and the supernatant was discarded. Then, 10% (vol/vol) of Acid-Citrate-Glucose (ACD) buffer (39 mM citric acid; 75 mM sodium citrate; 135 mM D-glucose, pH 4.5) was added, the PLTs were pelleted for 10 min at 3,000 rpm, and then resuspended to 50×10^8^ cells/mL in phosphate-buffered saline (PBS) buffer, as described [25], [26].

### Endothelial cell culture

Human umbilical vein endothelial cells (HUVECs, #KE-4109) were purchased from Kurabo (Osaka, Japan). The cells were used between passages 4 and 7 for experiments to avoid the effects of in vitro cell aging. They were grown in endothelial growth medium (HuMedia-EG2; Kurabo) supplemented with growth factors and 2% (v/v) fetal bovine serum to 70–80% confluency. They were cultured in a humidified incubator at 37°C with a 5% CO2 atmosphere, and the medium was changed every second day. To determine the effect of control and STZ PLTs on the cell signaling pathway, cells were cultured for 30 min in the presence of the control or STZ PLTs.

### Measurement of plasma parameters

Plasma parameters were measured as described previously [5]–[7], [23], [24]. We collected blood samples from non-fasting mice that had beeb euthanized by isoflurane overdose. Plasma samples were stored at −20°C until analysis. Briefly, plasma glucose, cholesterol, high-density lipoprotein (HDL) cholesterol, triglyceride, and serum non-esterified fatty acid (NEFA) levels were each determined with a commercially available enzyme kit (Wako Chemical, Osaka, Japan) by following the written instructions.

### Measurement of isometric force

The carotid artery was carefully isolated from a rat, dissected from the surrounding fat and connective tissue, cut into circular segments (2 mm long) and immediately placed in Krebs-Henseleit Solution (KHS) (composition in mM: NaCl: 118.0; KCl: 4.7; NaHCO_3_: 25.0; CaCl_2_: 1.8; NaH_2_PO_4_: 1.2; MgSO_4_: 1.2; glucose: 11.0). The vascular rings were mounted between two stainless-steel triangles in an organ bath containing KHS (37°C, pH 7.4) and aerated with 95% O_2_ and 5% CO_2_. The rings were stretched until a resting tension of 1 g was loaded, which was optimal for inducing maximal contraction. After 1-hr equilibration, the rings were contracted to a stable tension using PE (10^−6^ M). At the beginning of each experiment, the functional integrity of the endothelium was examined by precontraction of an isolated carotid artery with a submaximal concentration (EC_50_-EC_70_) of PE, followed by the addition of ACh (10^−6^ M). Concentration-response curves for ACh (10^−9^–10^−5^ M) and SNP (10^−10^–10^−5^ M) were obtained in a cumulative fashion using PE-precontracted arteries. Some rings were preincubated with L-NNA (10^−4^ M) 30 min before precontraction, when the effects of inhibitors on the responses to the above relaxant agents were to be examined. The effect induced by each concentration of ACh or SNP is expressed as a relaxation percentage of PE-induced precontraction. The results are expressed as the means ± SE, and n refers to the number of experiments.

### Measurement of NO production

NO detection (nitrite + nitrate) was performed as previously described [5], [6]. Each carotid artery was cut into transverse rings of 4 mm in length. These were placed in KHS at 37°C and then treated with ACh (10^−6^ M) for 15 min. The amount of NOx was calculated as follows: ACh-stimulated NOx [10^−5^ mol/min/g (weight of the carotid artery)]. The concentrations of nitrite plus nitrate (NOx) in the KHS and NOx standard (Eicom, Kyoto, Japan) were measured using an automated NO detector/high-performance liquid chromatography system (ENO20; Eicom).

### Measurement of TXB_2_ and 8-isoprostane levels in carotid artery

Each carotid arterial ring was placed for 10 min in a siliconized tube containing KHS at 37°C, and then 10^−6^ M ACh or vehicle (water) was applied for 15 min. Next, after the carotid arterial rings had been removed, the tubes were freeze-clamped in liquid nitrogen and stored at −80°C for subsequent analysis.

Thromboxane release was measured as in our previous studies [2]. TXB_2_, a metabolite of TXA_2_, was measured using a commercially available enzyme immunoassay kit (Cayman Chemical, Ann Arbor, MI, USA). The amount of TXB_2_ is expressed in pictograms per milligram wet weight of the carotid artery.

The measurement of 8-isoprostane was carried out with a commercially available enzyme-linked immunosorbent assay kit (Cayman Chemical, Ann Arbor, MI, USA).

### Measurement of superoxide anions

Carotid arteries were incubated with NBT to allow the superoxide generated by the tissue to reduce the NBT to blue formazan. Carotid arteries from controls treated with vehicle (saline), Control or STZ PLTs, and STZ-induced diabetic rats were cut into transverse rings of 5 mm in length. These were placed for 120 min at 37°C in 500 µL of KHS containing NBT (10^−4^ M). The NBT reduction was stopped by the addition of 0.5 N HCl (500 µL). After this incubation, the rings were minced and homogenized in a mixture of 0.1 NaOH and 0.1% SDS in water containing 40 mg/L of diethylentriaminepentaacetic acid. The mixture was centrifuged at 16,000 g for 30 min, and the resultant pellet was resuspended in 250 µL of pyridine at 80°C for 60 min to extract formazan. The mixture was then subjected to a second centrifugation (at 10,000 g for 10 min). The absorbance of formazan was determined spectrophotometrically at 540 nm. The amount of NBT reduced ( = quantity of formazan) was calculated as follows: amount of NBT reduced  =  A × V/(T × W × ε × l), where A is the absorbance, V is the volume of pyridine, T is the time the rings were incubated with NBT, W is the blotted wet weight of the aortic rings, ε is the extinction coefficient (0.7 L/mmol/mm), and l is the length of the light path. The results are reported in pmol/min/mg tissue.

### Cell extracts for Western blot analysis

Cells were seeded, incubated, and used as stated above. Cells in a confluent state were used for the tests. They were washed with PBS after the media were removed. Then, RIPA Buffer (Thermo Scientific, USA) containing protease inhibitor (Roche Applied Science) was added to the cells and they were gently harvested. Cell lysates were prepared and the protein content was quantified using the Pierce BCA Protein Assay Kit (Thermo Scientific). Lysates were then subjected to Western blot analysis.

### Western blotting

Each frozen sample was homogenized as described previously [2], [5], [6], [8], [23], [24] or stated above. Blots were performed as reported [2], [5], [6], [8], [23], [24]. To investigate the expressions of phospho-eNOS and phospho-Akt in such arteries upon ACh stimulation, carotid arterial rings from a given rat were incubated with KHS at 37°C and then exposed to 10^−6^ M ACh or vehicle (water) for 15 min. For the examinations of eNOS and Akt expression, we employed tissues not used for drug-treatment experiments. Carotid arterial protein extracts (20 µg) were applied to 10% SDS-PAGE and transferred to polyvinylidene difluoride membranes. Blots were incubated with anti-phospho-eNOS (Ser^1177^) (140 kDa; 1∶500), anti-eNOS (140 kDa; 1∶1,000), anti-phospho-Akt (Ser^473^) (60 kDa; 1∶1,000), anti-Akt (60 kDa; 1∶1,000), CD61 (100 kDa; 1∶1,000), CD62P (84 kDa; 1∶1,000) or anti-β-actin (42 kDa; 1∶5,000) antibodies, with detection being achieved using HRP-conjugated IgG followed by enhanced chemiluminescence. The band intensity was quantified by densitometry. The results were normalized to β-actin expression. To assess ACh-induced eNOS phosphorylation (at Ser^1177^) and Akt phosphorylation (at Ser^473^), we calculated the ratio of the optical density of phosphorylated eNOS or total eNOS, phosphorylated Akt, or total Akt in ACh-stimulated or nonstimulated (basal) samples in each case to that of the corresponding β-actin band. These values are presented as the fold increase in phosphorylated eNOS from the basal condition. In some experiments involving the detection of phospho-eNOS (Ser^1177^), phospho-Akt (Ser^473^), eNOS, Akt, and β-actin proteins, the same membranes were stripped.

### Monomer and dimer Western blotting

Low-temperature SDS-PAGE was performed to detect eNOS monomers and dimers. Briefly, cells lysates were prepared as above-stated. Protein lysates were resolved using a 6% Tris-glycine gel under reducing conditions. All gels and buffers were pre-equilibrated to 4°C before electrophoresis, and the buffer tank was placed in an ice bath during electrophoresis to maintain the gel temperature below 15°C. Standard blotting techniques were used, and membranes were incubated with mouse anti-eNOS polyclonal antibody as described above.

### Data analysis

Experimental vasorelaxation values are expressed as a percentage of the maximal contraction induced by PE in a given segment. Concentration-response curves were fitted using a nonlinear interactive fitting program (Graph Pad Prism 6.0; GraphPad Software, San Diego, CA, USA). Data are expressed as the mean ± SE, and n represents the number of rats. Statistical analysis was performed by 1- or 2-way analysis of variance (ANOVA) with a *post hoc* Bonferroni test or Student's *t*-test. *P*-values less than 0.05 were considered significant.

## Results

### Characteristics of STZ-induced diabetic rats

The characteristics of STZ-induced (diabetic) and Wistar (control) rats are summarized in Table 1. The body weight was lower, and blood glucose, cholesterol, triglycerides, and NEFA were higher in diabetic compared with control rats.

**Table 1:** Values of various parameters in diabetic and control rats.

Parameters	Control (5)	Diabetic (4–5)
Body weight (g)	**606.3±25.9**	337.5±18.6 ***
Glucose (mg/dL)	**123.6±6.5**	557.0±4.0 ***
Cholesterol (mg/dL)	**72.8±5.0**	111.7±6.6 **
HDL (mg/dL)	**52.9±2.4**	**51.7±2.8**
Triglycerides (mg/dL)	**139.8±26.0**	473.0±72.2 **
NEFA (mEq/L)	**0.22±0.04**	0.59±0.11 **

Values are means ± SE. Number of determinations is shown within parentheses. **P<0.01, ***P<0.001 vs. Control.

HDL, High Density Lipoprotein.

NEFA, non-esterified fatty acid.

### Relaxation responses to ACh and SNP in carotid artery

**Figure 1:**
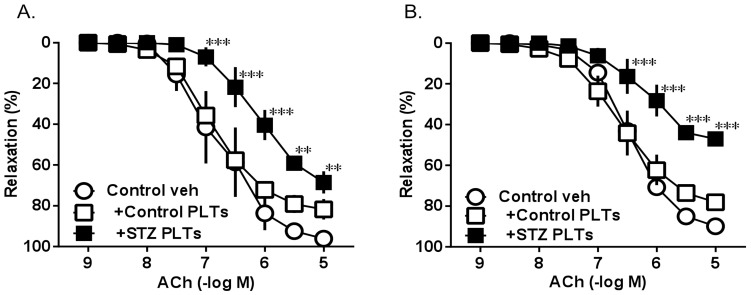
ACh-induced vasorelaxation of carotid arterial rings from controls induced for 30 min with PLTs isolated from control (Control PLTs) and diabetic (STZ PLTs) rats. (A) 36-weeks old. (B) 52-weesks old. Carotid arterial rings were preincubated with each type of PLT (1.65×10^7^ cells/mL+ 30 min). Data are means ± SE; n = 4; **P<0.01, ***P<0.001 vs. control.

The experiments described here were performed 28–44 weeks after the injection because carotid arteries from young STZ-induced diabetic rats (36 weeks old) and STZ-induced chronic diabetic rats (52 weeks old) exhibited similar ACh-induced relaxation (Fig. 1).

**Figure 2:**
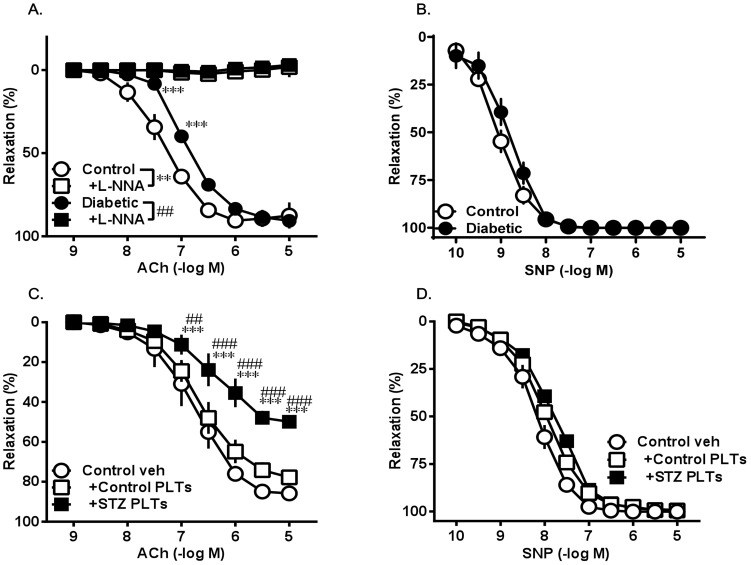
Concentration-response curves for endothelium-dependent vasorelaxation elicited by the cumulative application of ACh (A and C) and endothelium-independent relaxation by incremental concentrations of SNP (B and D) in isolated carotid arterial rings. (A) Effects of the NOS inhibitor L-NNA on the ACh-induced relaxation of carotid arterial rings from control and diabetic (STZ-induced) rats. Carotid arterial rings were preincubated with L-NNA (10^−4^ M; 30 min). (C and D) Effects of PLTs. ACh-induced (C) and SNP-induced (D) vasorelaxation of carotid arterial rings from controls induced for 30 min with PLTs isolated from control (Control PLTs) and diabetic (STZ PLTs) rats. Carotid arterial rings were preincubated with each type of PLT (1.65×10^7^ cells/mL; 30 min). Data are means ± SE; n = 5; ***P<0.001 vs. Control, *##P*<0.01 or ###*P<*0.001 vs. Diabetic or +Control PLTs.

The administration of ACh in cumulative concentrations for the activation of Akt/eNOS induced endothelium-dependent vasorelaxation, which was impaired in the presence of diabetes (Fig. 2A). In diabetic and control rats, the above relaxation responses were abolished by treatment with the NOS inhibitor L-NNA.

The concentration response curve for the NO donor SNP, which was used to assess endothelium-independent vasorelaxation, was similar between diabetic and control rats (Fig. 2B).

### PLTs impair endothelium-dependent vasorelaxation

Carotid arterial rings were incubated for 30 min with PLTs isolated from control (control PLTs) and STZ-induced diabetic (STZ PLTs) rats to study the effects on ACh- and SNP- induced vasorelaxation. The basal tone of the carotid arterial rings showed no significant change in any of the experimental groups. The relaxation response to ACh but not to SNP markedly diminished in carotid arterial rings preincubated with STZ PLTs (Fig. 2C and 2D). This suggests that STZ PLTs selectively affect the endothelium, as relaxation responses to ACh and SNP are endothelium-dependent and -independent, respectively. The impaired vasorelaxation in response to ACh in carotid arterial rings treated with STZ PLTs could not be attributed to an impaired responsiveness of vascular smooth muscle cells to NO, because vessels from all experimental groups were equally responsive to the NO donor SNP.

### STZ PLTs reduce NO production and increase TXA_2_ in carotid artery

**Figure 3:**
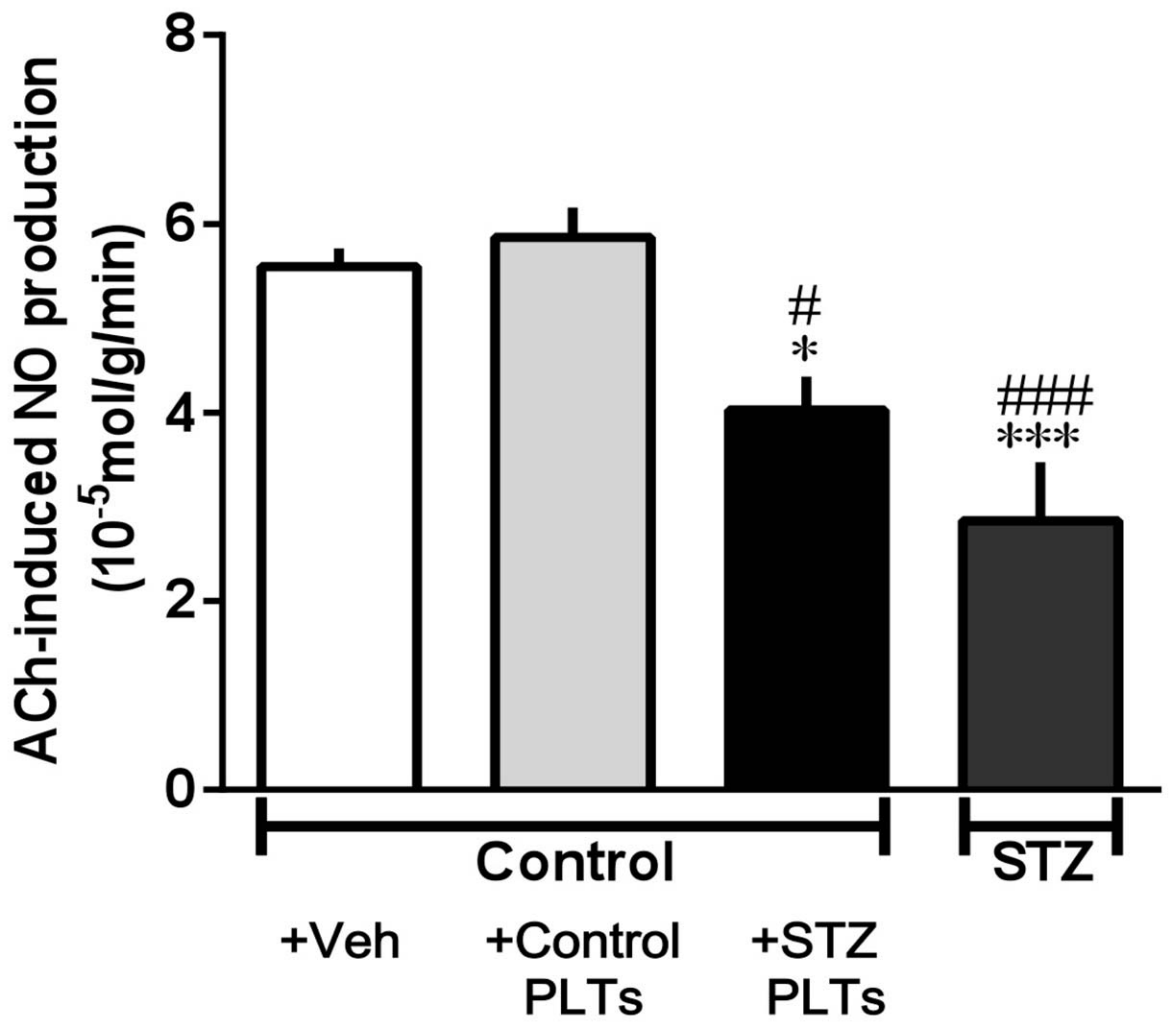
Release of NOx under ACh stimulation in carotid arteries. Control carotid arteries were incubated for 30°C with 1.65×10^7^ cells/mL of control or STZ PLTs before this experiment. The release of NOx in each treated or untreated carotid artery from control or diabetic rats with ACh (10^−6^ M) stimulation. Data are means ± SE; n = 5–6. *P<0.05 or ***P<0.001 vs. Control+Veh (only ACh-treated carotid arteries from control). #*P<*0.05, ##*P<*0.01, or ###*P<*0.001 vs. Control + Control PLTs.

To assess NO production by carotid arterial rings, changes in the NO concentration in response to ACh (10^−6^ M) were investigated. ACh-induced NO release was diminished in carotid arterial rings pretreated with STZ PLTs and carotid arterial rings isolated from STZ-induced diabetic rats but not in carotid arterial rings pretreated with control PLTs (Fig. 3).

**Figure 4:**
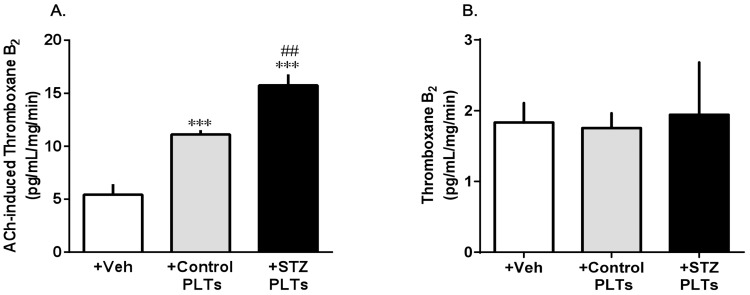
Release of TXB_2_ (stable metabolite of TXA_2_) under non-stimulation (A) or ACh-stimulation (B) in carotid arteries. Control carotid arteries were incubated for 30°C with vehicle (saline) or 1.65×10^7^ cells/mL of control or STZ PLTs before this experiment. (B) TXB_2_ production in each treated or untreated carotid artery from control or diabetic rats with ACh (10^−6^ M) stimulation. Data are means ± SE; n = 5–6. ***P<0.001 vs. Control+Veh (only ACh-treated carotid arteries from control). ##*P<*0.01 vs. Control + Control PLTs.

To examine whether PLTs only decrease NO production, we assessed the production of TXB_2_, a metabolite of TXA_2_, on stimulation with ACh (10^−6^ M) in carotid arterial rings pretreated with STZ and control PLTs (Fig. 4A). The levels of TXB_2_ release stimulated by ACh increased in carotid arterial rings treated with STZ PLTs relative to those treated with control PLTs (Fig. 4A). Whereas the TXB_2_ levels were equal in all groups (Fig. 4B).

### PLTs induced Akt/eNOS inactivation and expression of eNOS

**Figure 5:**
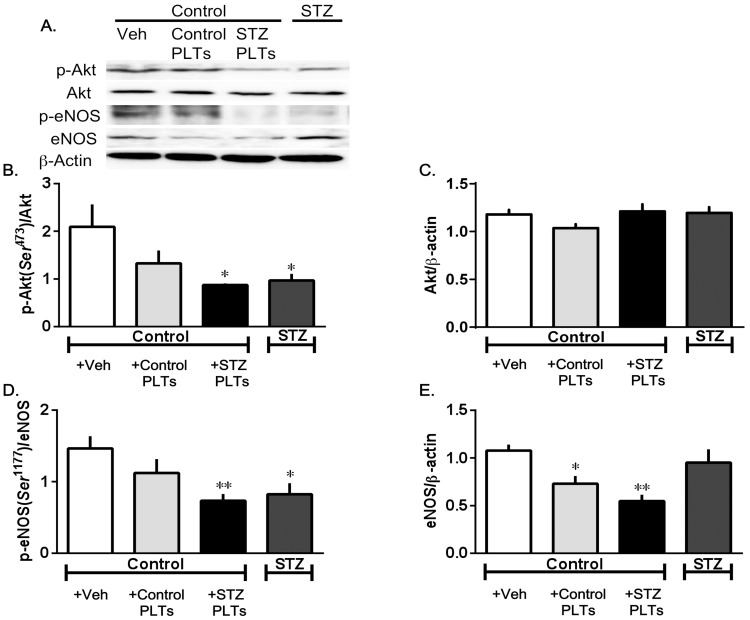
Effect of PLTs on the phosphorylation of Akt and eNOS. (A) Representative Western blots. Single bands at 60 kDa (Akt), 140 kDa (eNOS), and 42 kDa (β-actin) were observed. (B and D) Effect of PLTs isolated from control or diabetic rats (1.65×10^7^ cells/mL; 30 min) on ACh-induced Akt phosphorylation (Ser^473^) (B) or eNOS hosphorylation (Ser^1177^) (D). (C) Total Akt and eNOS expression. (E) Total eNOS expression. Data are means ± SE; n = 5–6. *P<0.05 or **P<0.01 vs. *+Veh (ACh-stimulated carotid artery from untreated control).

**Figure 6:**
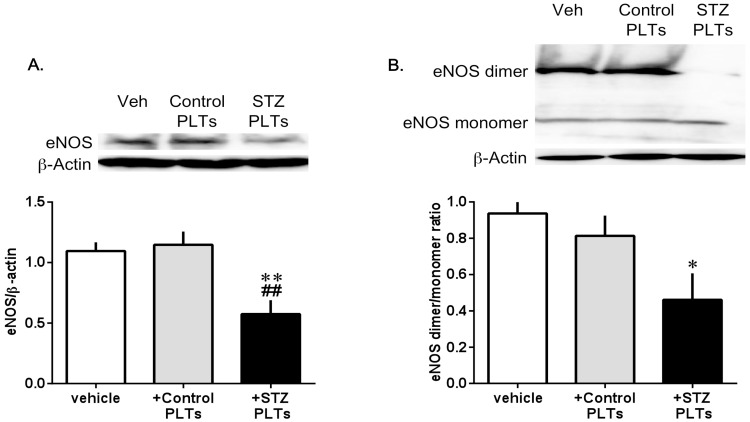
Total eNOS and eNOS dimers/monomers in HUVEC cultured under control PLTs or STZ PLTs stimulation. Western blots for eNOS expression (A) and eNOS dimer/monomer (B) in HUVECs cultured with PLTs (1.65×10^7^ cells/mL; 30 min) isolated from control or diabetic rats. Data are means ± SE; n = 6; *P<0.05, **P<0.01 vs. vehicle, ##*P<*0.01 vs. +Control PLTs.

The suppression of ACh-induced activation of the Akt/eNOS pathway by STZ PLTs was indicated by significant decreases of Akt and eNOS phosphorylations in carotid arterial rings incubated with 1.65×10^7^ cells/mL of STZ or control PLTs for 30 mins. STZ but not control PLTs reduced the phosphorylation of Akt on Ser 473 kinase involved in eNOS phosphorylation, without its expression (Fig. 5A and 5B). Also, treatment with STZ but not control PLTs led to a significant decrease in eNOS phosphorylation on the activator (Ser 1177) site (Fig. 5A and 5D). eNOS expression was significantly reduced after control and STZ PLT treatment (Fig. 5A and 5E). However, since this result was surprising, it was confirmed using HUVECs. There was a significant decrease in the expression of HUVECs treated with STZ PLTs (Fig. 6A). Furthermore, we also measured the expression of eNOS in the dimer/monomer ratio, and showed that there was a significant reduction in the eNOS dimer/monomer ratio in HUVECs treated with STZ PLTs (Fig. 6B). These data suggest that STZ PLTs play a significant role in the development of endothelial dysfunction.

### PLT activation

**Figure 7:**
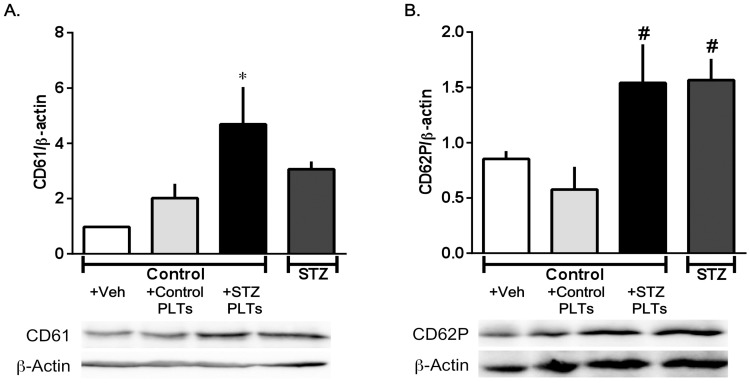
CD61 (A) and CD62P (B) expression in carotid arterial strips from controls. Western blots for CD61 or CD62P in carotid arterial strips from controls treated with PLTs (1.65×10^7^ cells/mL; 30 min) isolated from control or diabetic rats. Data are means ± SE; n = 5–6; *P<*0.05 vs. *+Veh (carotid artery from untreated control) and #+Control PLTs (carotid artery from control treated with control PLTs).

To further investigate the mechanism of Akt/eNOS pathway impairment after treatment with PLTs, we performed Western blotting with the PLT marker CD61. As shown in Fig. 7A, in the carotid artery treated with STZ PLTs, a very strong CD61 signal was observed, with little to distinguish between the carotid arteries treated with STZ PLTs and those isolated from STZ-induced diabetic rats. These data suggest that many PLTs isolated from STZ-induced diabetic rats adhere to the carotid artery.

The extent of in vivo PLT activation was measured by analysis of the surface expression of P-selectin as a marker of PLT degranulation (CD62P, Fig. 7B) in the carotid artery after treatment with STZ or control PLTs. CD62P levels were significantly increased in carotid arteries treated with STZ PLTs and those from STZ-induced diabetic rats, suggesting that, in this experiment, STZ PLTs were present on the carotid artery, an effect that is likely attributable to the activated PLT function.

### Vascular oxidative stress

**Figure 8:**
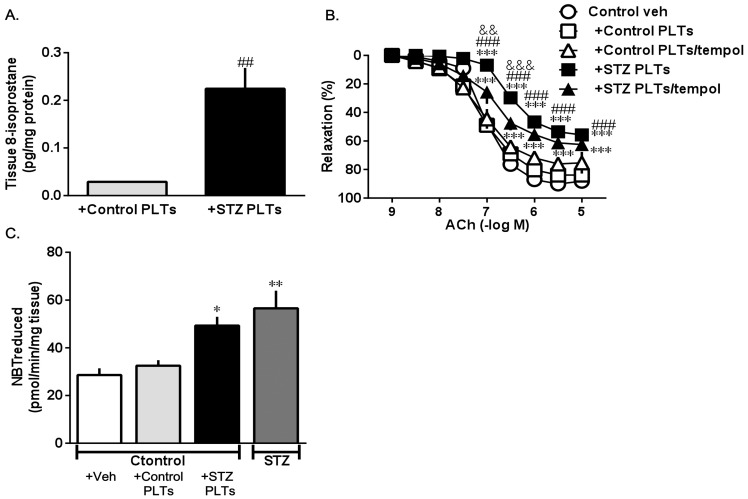
8-isoprostane concentrations in carotid arterial strips from controls treated with control or STZ PLTs, A. Data are means ± SE; n = 5; *##P<*0.01 vs. +Control PLTs. Effect of the SOD mimetic tempol on the ACh-induced relaxation of carotid arterial rings from controls induced for 30 min with PLTs isolated from control (Control PLTs) and diabetic (STZ PLTs) rats and tempol (10^−4^ M), B. Carotid arterial rings were preincubated with each type of PLT (1.65×10^7^ cells/mL; 30 min) or each type of PLT along with tempol. Data are means ± SE; n = 4, ***P<0.001 vs. Control veh, ###*P<*0.001 vs. +Control PLTs, &&*P<*0.01 or &&&*P<*0.001 vs. +STZ PLTs/tempol. Quantification of carotid arterial superoxide anion production by measuring the amount of reduced NBT, C. Control carotid arteries were incubated for 30 min at 37°C with 1.65×10^7^ cells/mL of Control or STZ PLTs. Data are means ± SE; n = 5; *P<0.05 or **P<0.01 vs. control + Veh.

Increased 8-isoprostane is a recognized marker of oxidative stress [27]. To investigate whether PLTs can induce oxidative stress in carotid arteries, the levels of 8-isoprostane were analyzed. As demonstrated in Fig. 8A, carotid arteries treated with STZ PLTs showed a clearly increased level of 8-isoprostane, suggesting that STZ PLTs generate oxidative stress.

Furthermore, we examined the effect of the SOD mimetic tempol on ACh-induced relaxation. In rat carotid arterial rings, cotreatment with tempol and STZ PLTs increased ACh-induced relaxation (Fig. 8B). This suggests that, in rat carotid arterial rings, exposure to STZ PLTs may lead to the excessive generation of superoxide which may, in turn, trigger an impairment of endothelium-dependent relaxation via the Akt/eNOS signaling pathway.

## Discussion

In the present study, we demonstrated that activated PLTs impair the endothelial function and increase levels of oxidative stress, suggesting that decreased NO bioavailability and increased vasoconstriction contribute to the effects of PLTs in diabetes. It is particularly interesting to note that the impairments were mainly due to STZ PLTs. We also demonstrated that STZ PLTs decreased NO production by carotid arteries, which was linked to a decrease in phosphorylations of Akt and eNOS at the stimulatory site. Taken together, these results suggest that STZ PLTs induce endothelial dysfunction and highlight that PLTs from diabetic rats affect carotid arteries mainly via the NO pathway and oxidative stress.

Endothelial dysfunction is a common feature in cardiovascular diseases characterized by the reduced synthesis or altered activity of vasodilative mediators, such as NO, and increased effects of vasoconstrictors such as TXA_2_ [28]. In the present study, we observed the dysfunctional relaxation of carotid arteries in STZ-induced diabetic rats, as shown by decreased ACh-induced vasorelaxation, suggesting that diabetes induces dysfunctional vasorelaxation in carotid arteries. The pathological changes are characterized by impairments in endothelial integrity and vasorelaxation, consistent with previous studies in diabetic carotid arteries [29]. L-NNA abolished the relaxation induced by ACh in carotid arteries. This confirms the predominance of NO as an endothelium-derived relaxing factor in carotid arteries. SNP, an NO donor, induces vascular smooth muscle relaxation and, the smooth muscle layer did not display discernible changes in SNP-induced function. In addition, we showed that STZ PLTs are able to induce endothelial dysfunction in control carotid arterial rings, indicating that the effects induced by STZ PLTs are independent of the number of PLTs but dependent on the different compositions and/or origin of control and STZ PLTs.

The expression of eNOS protein or eNOS phosphorylation in the endothelium has been shown to play a critical role in vasorelaxation, because the deletion of eNOS or decreased eNOS activity causes dysfunctional vasorelaxation in most vessels. When Ser1177 is phosphorylated by several kinases, such as Akt, NO production is increased to two or three times basal levels [9]. Enzymatic activity of eNOS is regulated by multiple phosphorylations of specific sites on the eNOS protein. The most well-studied are the functional consequences of the phosphorylation of Ser1177 and Thr495. Ser1177 is a positive regulatory site of eNOS, and Thr495 is a negative regulatory site of eNOS in that phosphorylation leads to increased or decreased enzymatic activity. It has been reported that insulin and ACh can activate eNOS phosphorylation on Ser1177, and that these activations occur via serine/threonine protein kinases, such as Akt (5). Similarly, CaMKII reportedly enhances eNOS phosphorylation on Ser1177 via the activation of serine/threonine protein kinases, such as Akt (5). So, we examined the eNOS phosphorylation on Ser1177 and Akt phosphorylation on Ser473 under ACh stimulation. We found that STZ PLTs were able to modify eNOS phosphorylation. Indeed, STZ PLTs decreased Ser1177 phosphorylation in carotid arteries. Furthermore, STZ PLTs decreased Akt phosphorylation, with Akt being the kinase involved in eNOS activation-associated phosphorylation in the carotid artery [10], [30]. In fact, the present data suggest that the STZ PLTs mediated reduction in ACh-induced NO production in carotid arteries, and decreased eNOS activity probably acts to reduce NO production in carotid arteries. In addition, we also showed that treatment with STZ PLTs decreased the expression of eNOS. Thus, PLTs may affect endothelial NO vasodilatation by altering eNOS expression (total eNOS protein and eNOS dimer/monomer ratio). Moreover, the result of treating HUVECs with STZ PLTs was the same as the result for carotid arterial rings (Fig. 5E). So, we suggest that the endothelial cells in the carotid artery have a large number of contacts with PLTs. In this respect, rats vessels treated with PLTs displayed a marked reduction in eNOS expression, suggesting that PLTs directly impair the endothelial function, most likely through reducing both the total eNOS expression (total expression and dimerization) and eNOS activity. However, the mechanisms by which STZ PLTs act on the carotid artery to cause reductions in eNOS expression require further investigation.

Abnormalities in TXA_2_ production were among the earliest characterized abnormalities in PLTs of diabetic subjects [31]. Vascular overproduction of endothelium-dependent contraction factors, including TXA_2_, has been reported to cause endothelial dysfunction in several arteries from diabetic models [2], [31]. Consequently, we decided to examine the release of TXB_2_, a metabolite of TXA_2_, in carotid arteries treated with control and STZ PLTs. TXA_2_, a lipid mediator originating from arachidonic acid metabolism through the cyclooxygenase (COX) pathway, is a powerful constrictor of vascular smooth muscle. Another study performed by our laboratory revealed that the vascular production of TXA_2_ was increased in diabetes, and that inhibition signaling improved endothelial dysfunction (8). We found that ACh stimulation only increased TXB_2_ levels in carotid arteries treated with control and STZ PLTs, suggesting that ACh stimulates TXA_2_ production. However, it is not clear how ACh controls the production of TXA_2_. In this context, further research is needed.

A variety of adhesion molecules are pre-stored in intraplatelet granules and expressed on the PLT surface on activation. The glycoprotein (GP) IIIa, also known as integrin β3 (CD61), is the main receptor mediating PLT aggregation and the most abundant receptor expressed on the PLT surface [32], [33]. A clear corollary to this important finding is that PLTs adhere to the endothelial surface. Surprisingly, we revealed an increase in CD61 expression in carotid arteries treated with STZ PLTs, which may indicate that PLTs adhere to endothelial cells in the carotid artery. PLTs in diabetic patients show increased expression of CD61 [34]. Increased expression of CD61 on PLT surfaces leads to enhanced fibrinogen binding and, subsequently, PLT cross-linking and thrombogenesis [35]. It must be noted that fibrinogen levels can be raised in association with diabetes [31]. Furthermore, we found that CD62P, known as P-selectin, a marker of activated PLTs, was increased in carotid arteries treated with STZ PLTs. P-selectin is a cell adhesion molecule that translocates to the PLT surface upon activation. P-selectin is responsible for the adhesion of certain leukocytes and PLTs to the endothelium, and the plasma concentration of soluble P-selectin is now recognized as a predictor of adverse cardiovascular events [36]. Additionally, markers of PLT activation were already found to have increased in individuals positive for islet cell antibodies before the onset of overt diabetes mellitus, indicating that PLT activation occurs very early during the development of diabetes [37]. This is clinically reflected by the fact that patients with diabetes without prior cardiovascular events have a risk of myocardial infarction similar to that among non-diabetic patients with prior myocardial infarction. Thus, activated PLTs have a major impact on morbidity and mortality, as most diabetic patients die from cardiovascular atherothrombotic events [38].

Impaired endothelial function has been described in very early stages of diabetes mellitus and hyperglycemia, and decreased insulin-sensitivity, as well as increased oxidative stress, have been proposed as possible contributors [4], [12], [39], [40]. Increased oxidative stress is widely accepted as involved in the development and progression of diabetes and endothelial dysfunction via the PI3-K/Akt pathway and eNOS dimer/monomer ratio. On the other hand, it is well known that activated PLTs on vascular walls are important sources of reactive oxygen species, such as superoxide anions [22]. We assessed superoxide generation by measuring the amount of NBT reduced by superoxide. The basal superoxide level was greater in carotid arteries from STZ-induced diabetic rats and control rats treated with STZ PLTs than in those from control rats (please see drawing below). Previous studies suggested that oxidative stress plays a major role in the impairment of endothelium-dependent responses that occur in diabetes [24]. Vascular homeostasis is dependent on the balance between dilatation (such as NO production by eNOS activity) and constriction (such as TXA_2_ production). Furthermore, we found higher levels of isoprostane in carotid arteries treated with STZ PLTs and lower levels of eNOS dimer in HUVECs compared to those treated with control PLTs under non-stimulation conditions. Thus, abnormal PLT activation in the diabetic state may be a cause of excessive oxidative stress. As shown in Fig. 2B, ACh stimulation increased TXB_2_ levels in carotid arteries treated with STZ PLTs. Several recent studies demonstrated that oxidative stress in endothelial walls reduced the phosphorylation of Akt/eNOS and increased ACh-induced TXA_2_ production, resulting in impaired endothelium-dependent relaxation. Therefore, our results suggest that STZ PLT-induced oxidative stress inhibited the activation of the Akt/eNOS signaling pathway and increased ACh-induced TXA_2_ production in the endothelium of the carotid arteries. Our finding that tempol, a cell-permeable scavenger of superoxide, partially restored vascular responses in diabetic rats is consistent with the above idea of a crucial role in oxidative stress.

**Figure 9:**
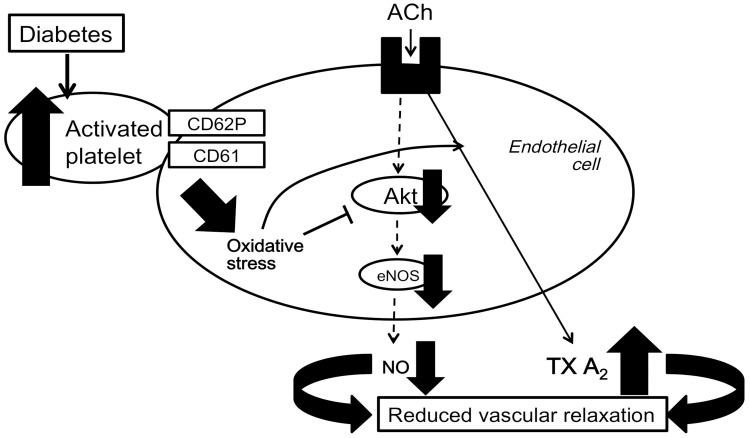
Role of PLT activation in the relationship between diabetes and reduced vascular relaxation.

From the above, our data are consistent with the following scenarios (Fig. 9): STZ-induced diabetic rats have increased levels of activated PLTs. STZ PLTs adhere to endothelial cells in the carotid artery and increase oxidative stress. STZ PLTs can impair the endothelial function in rat carotid arteries, at least partly, by directly reducing Akt and eNOS activity (via decreasing total eNOS protein and eNOS dimerization), and increasing TXA_2_ production. Taken together, these data strongly suggest that circulating diabetic PLTs induce endothelial dysfunction, and this model demonstrates for the first time their pathophysiological significance. From these results, one can advance the hypothesis that diabetic PLTs contribute to the pathophysiological process of diabetic endothelial dysfunction.
